# Exploring gene signatures and regulatory networks in a rat model of sciatica: implications and validation in neuropathic pain

**DOI:** 10.3389/fnmol.2023.1261217

**Published:** 2024-02-06

**Authors:** Mu Xu, Zhijian Wang, Gang Xu, Mengye Zhu, Daying Zhang, Yi Yan

**Affiliations:** Department of Pain Medicine, The First Affiliated Hospital, Jiangxi Medical College, Nanchang University, Nanchang, Jiangxi, China

**Keywords:** neuropathic pain (NP), RNA sequencing (RNA-Seq), differentially expressed genes (DEGs), chronic systolic injury of sciatic nerve, sciatica

## Abstract

**Background:**

Sciatica (neuropathic pain [NP]) is a common disease characterized by pain from radiation along the sciatic nerve. The aim of this study was to study the genes associated with chronic systolic injury of sciatic nerve (SCN-CCI) in rats by RNA-Seq technique, and to explore their potential as therapeutic targets.

**Methods:**

Sciatic nerve rat model was obtained by ligation of sciatic nerve and divided into two groups: SCN-CCI group and Sham group. Behavioral assessments were performed to evaluate pain sensitivity, following which their spinal cord dorsal horn were resected and RNA sequencing was conducted to identify differentially expressed genes (DEGs). Bioinformatics and functional enrichment analysis was performed to identify promising DEGs and their related biological processes and pathways associated with SCN-CCI. PPI network analysis and hub gene identification were conducted. QRT-PCR, western blot, ELISA, and immunofluorescence staining were performed on rat models to validate the expression of these hub genes and investigate related proteins and inflammatory markers.

**Results:**

The SCN-CCI rat model was successfully obtained, exhibiting increased pain sensitivity compared to the Sham group, as indicated by decreased mechanical allodynia thresholds, thermal latencies, and increased paw withdrawals. RNA-Seq analysis identified 117 DEGs in the SCN-CCI rat model, involved in various biological processes and pathways related to sciatica. PPI network analysis revealed hub genes, including Ly6g6e, which exhibited significant differential expression. QRT-PCR and Western blot analysis confirmed the expression patterns of these hub genes. Pain behavior assessment demonstrated reduced pain thresholds and increased paw flinching responses in the SCN-CCI group. Furthermore, the SCN-CCI group showed upregulated expression of Ly6g6e, increased protein levels of Ly6g6e, CGRP, and NGF, as well as elevated levels of IL-1β, MCP-1, and IL-6, and microglial cell activation in the spinal dorsal horn. ELISA results confirmed the increased levels of IL-1β, MCP-1, and IL-6 in the spinal dorsal horn.

**Conclusion:**

These comprehensive findings provide valuable insights into the SCN-CCI rat model, DEGs associated with sciatica, hub genes (Ly6g6e as promising targets), pain behavior changes and molecular alterations.

## Introduction

Neuropathic pain (NP) is chronic secondary pain arising from primary lesions, dysfunction, or temporary disturbances in the peripheral or central nervous system (Finnerup et al., 2021). It affects around 7–10% of the global population, with a higher prevalence among individuals aged 50 and above. NP can be categorized into peripheral and central NP based on the location of the underlying lesion (Bouhassira, 2019). Unlike other nociceptive pains, NP is characterized by persistent or intermittent spontaneous pain, often described as burning, shooting, stabbing, needle-like, crushing, or freezing (Colloca et al., 2017). This condition imposes both physical and psychological stress on patients. However, the specific mechanisms and causative factors of NP remain unknown, making it challenging to find a cure. Currently, tricyclic antidepressants (TCA), gabapentin and pregabalin are the main recommended therapeutic drugs for NP in clinical practice and primarily work by inhibiting the calcium and sodium channels in nerve cells. Unfortunately, their effectiveness is not always satisfactory, providing only partial relief or ineffective for a significant number of patients due to tolerance and can be accompanied by side effects (Mendlik and Uritsky, 2015; Bates et al., 2019). Thus, further research is needed to find new targets to improve treatment outcomes.

With the development of biotechnology, the mechanism of NP has been further explored based on genes, non-coding RNAs and epigenetic modifications to look for molecular targets and therapeutic strategies of better efficacy. Studies have shown that spinal excitatory neurons expressing protein kinase C-γ in inner layer of laminae II are involved in pathological pain (Peirs et al., 2015), and both the TRP channel family and Toll-like receptors are involved in this process (Xiao Y. et al., 2019). Additionally, epigenetic regulation has also been discovered to have a close link with NP development. Moreover, DNA methyltransferases (Dnmt1 and Dnmt3) can induce hyperexcitability by regulating the expression of potassium channel Kcna2 in peripheral neurons after nerve injury (Descalzi et al., 2015). Also, some key genes are involved in the relief or exacerbation of NP. For example, PKM2 was found to be highly expressed in the spinal cord of rats with chronic constriction injury (CCI)-induced NP in a study by Wang et al. (2018) and inhibition of PKM2 expression effectively attenuated the pain and inflammatory response in rats. Wu et al. (2020) revealed that AQP9 knockdown promoted the development of NP. Furthermore, as reported by Wu et al., miR-30d-5p, miR-125b-5p, and miR-379-5p could regulate the expression of key mediators in NP, such as TNF-α, Stat-3 and BDNF (Wu et al., 2020). As researches go deeper, some key genes and epigenetic mechanisms related to NP have been gradually discovered, but many are not.

To fill the literature gap in this field, we conducted a comprehensive study using rat models of sciatic nerve chronic constriction injury (SCN-CCI) to simulate the condition in humans. Our aim was to unravel the molecular changes associated with NP and identify key genes involved in its development and progression. Using rat models of SCN-CCI and sham surgery, gene expression analysis was performed in the spinal cord dorsal horn tissues of both groups at different time points via RNA-sequencing, following which Ly6g6e and was identified and selected for further investigation. Then, bioinformatic analysis was conducted to gain insights into the biological functions and pathways related to the identified genes. To validate these results obtained, basic research techniques such as qRT-PCR and Western blot analysis were performed. Additionally, to explore the specific impact of Ly6g6e, experiments were conducted in the SCN-CCI + siNC group (negative control siRNA) and the SCN-CCI + si-Ly6g6e group (Ly6g6e siRNA) to assess the potential therapeutic effects of targeting Ly6g6e expression in the context of SCN-CCI-induced NP. Overall, these findings shed light on the functional role of the identified genes in NP and their potential as therapeutic targets.

## Materials and methods

### Construction of the animal model

Adult male Sprague Dawley (SD) rats (weight, 280–320 g; age, 6–8 weeks) were purchased from Beijing Vital River Laboratory Animal Technology Co., Ltd. The rats were housed under controlled conditions at 25 ± 2°C with a 12/12-h light/dark cycle and had free access to water and food throughout the study. Following a 1-week acclimation period. Measures were taken to minimize the number of animals used and their suffering during the experiment. The ethics committee of the First Affiliated Hospital of Nanchang University (CDYFY-IACUC-202305QR014) and was conducted in accordance with corresponding guidelines.

Two different rat models were constructed in this study. The first model comprised a SCN-CCI group (sciatic nerve underwent chronic constriction injury, *n* = 12 rats) and a sham group (underwent sham surgery, *n* = 12 rats). The SCN-CCI model of rats was obtained following the method described in the study by Zhao et al. (2020). The rats from the three groups were anesthetized with 2% pentobarbital sodium administered via intraperitoneal injection. For the SCN-CCI group, the left sciatic nerve in the middle of the thigh was exposed by blunt dissection of the muscles. The nerve was then freed from the adhesive tissue near the trifurcation of the sciatic nerve and was ligated at four sites with 1 mm intervals above the sciatic trifurcation branch, with the ligated portion of the nerve measuring approximately 4–5 mm in length. Of note, the tightness of the ligatures was adjusted to a suitable level where slight twitching of the surrounding muscles could be observed. Comparatively, for the Sham group, the left sciatic nerve was exposed without ligation. In the second model, the rats were randomly divided into three groups (*n* = 6 rats/group): SCN-CCI group (underwent chronic constriction injury, as described above), SCN-CCI + Ly6g6e group (after SCN-CCI modeling, the rats were given intrathecal injections of Ly6g6e siRNA), and the SCN-CCI + siNC group (control group). The SCN-CCI + siNC group received intrathecal injections of negative control siRNA (20 μM, 10 μL) for 3 days before surgery. In contrast, the SCN-CCI + si-Ly6g6e group received intrathecal injections of Ly6g6e siRNA (20 μM, 10 μL) for 3 days prior to surgery to specifically target Ly6g6e expression.

The rats (*n* = 2 each time point) were euthanized under pentobarbital sodium anesthesia and tissue samples from the left L4-L6 spinal segments were collected at different time points, including pre-operation (0 days) and post-operation at 3, 7, and 14 days.

*Behavioral assessments of pain were conducted on days 0, 3, 7, 10, and 14 using the following tests:*

*(i) Mechanical allodynia (Paw Withdrawal Mechanical Threshold, PWMT).* Mechanical allodynia in the left hind paws was evaluated using electronic von Frey filaments (IITC, Woodland Hills, CA) in rats from the Sham group, SCN-CCI group, SCN-CCI + siNC group, and SCN-CCI + si-Ly6g6e group. This evaluation helps measure the sensitivity of rats to mechanical stimuli. Briefly, the animals were habituated to the plexiglass chamber for 15 min 2 days before the experiment and each measurement. Next, von Frey filaments of increasing force ranging from 1 to 60 g were applied vertically to the plantar surface of the hind paw until the filament was slightly bent. The test was discontinued if a positive response was observed, indicating rapid paw withdrawal or flinching. The maximum test duration for each trial was set to 2 s. To ensure accuracy, each filament was tested repeatedly with 20-s intervals between trials. A total of 5 trials were performed. The tactile threshold was recorded as the force at which a positive response was elicited in at least three-fifths of the trials (Wang et al., 2018).

*(ii) Thermal hyperalgesia (Paw Withdrawal Thermal Latency, PWTL).* This was assessed in rats from the Sham group, SCN-CCI group, SCN-CCI + siNC group, and SCN-CCI + si-Ly6g6e group to determine the sensitivity of rats to thermal stimuli. Briefly, the time from the initiation of radiant-heat stimulation to the withdrawal response was measured using a 37370 plantar testing instrument (Ugo Basile, Shanghai Yuyan Scientific Instrument Co., Ltd., China). Thermal hyperalgesia was evaluated after a 10-min period of mechanical allodynia testing. In this procedure, the rats were placed inside a plastic chamber with a glass floor. A light source located beneath the glass floor was focused on the midsole surface of the hind paw. When the withdrawal reflex occurred, interrupting the light beam, the light and timer were automatically switched off. To prevent burns, the intensity of the light was adjusted to a constant rate of 70 IR, and a cut-off time of 30 s was set. The withdrawal latency of each paw was measured at 5-min intervals, and the average of three readings was recorded for further analysis (Wang et al., 2018).

*(iii) Cold allodynia.* Cold allodynia in the left hind paw was evaluated using the acetone test in rats from the Sham group, SCN-CCI group, SCN-CCI + siNC group and SCN-CCI + si-Ly6g6e group to measure the sensitivity of rats to cold stimuli, indicated by the number of paw withdrawals in response to cold stimulation. Briefly, the rat’s hind paws were positioned on a wire mesh for the test. Afterward, the rat’s response to acetone was observed and recorded. One minute after applying 100 μL of acetone onto the plantar surface of the hind paws, any paw withdrawal, shaking, or licking exhibited by the rat was documented as a pain response. The test was repeated five times for each rat, with 1-min intervals between each repetition (Abbaszadeh et al., 2018).

*(iV) Assessment of spontaneous pain behaviors.* The experiment was carried out according to the research method of Banik et al. (2023). In short, spontaneous leg lift behavior was measured in Sham group, CCI group, CCI + siNC group and CCI + si-Ly6g6e group at 0, 3, 7, 10, and 14 days, and the spontaneous foot-lifting (SFL) behavior including SFL duration (SFLd) and number (SFLn) were recorded (Banik et al., 2023).

### RNA extraction, library construction and sequencing

Total RNA was extracted from the tissue using Trizol reagent (15596018, Thermo Fisher, USA). The extracted RNA samples were then digested with RNase-free DNase I (EN0521, Invitrogen, USA) to remove any remaining genomic DNA. Following digestion, the resulting products were purified using magnetic beads (Axygen, USA). The concentration, quality, and integrity of the extracted total RNA were assessed using a NanoDrop 2000 spectrophotometer (Thermo Scientific, USA) and an Agilent 2100 Bioanalyzer (Agilent Technologies, USA).

For sequencing, total RNA samples with a standard concentration of ≥200 ng/μL, a quality score of ≥10 μg, and an RNA integrity number (RIN) of ≥8.0 were selected. Briefly, the mRNA from each sample was enriched using oligo(dT) magnetic beads and then fragmented into short fragments of approximately 200 bp in a fragment buffer. Subsequently, first-strand cDNA was synthesized from the fragmented RNAs using random hexamer primers, and second-strand cDNA was synthesized using a reaction mixture containing buffer, dNTPs, RNase H, and DNA polymerase I. The purified double-stranded cDNA was subjected to end repair using magnetic beads, followed by 3′ monoadenylation. The size and integrity of the treated cDNA were assessed using an Agilent 2100 Bioanalyzer. Finally, the cDNA libraries were sequenced on an Illumina HiSeq™ 4000 platform using a paired-end flow cell.

### Data cleaning and screening of differentially expressed genes

The raw counts obtained from sequencing were subjected to preprocessing using FastQC software. After preprocessing, which included the removal of PCR duplicates, reads containing adapters and poly-N sequences, and low-quality reads with a score of ≤5, the filtered data was utilized for subsequent analysis. Transcript expression levels were evaluated using the reads per kilobase per million (FPKM) values and quantified using RSEM software. FPKM values represent the expression of genes across different samples. Bioinformatic analysis was performed using the R software, with the “Edge” package utilized to screen NP-related DEGs in rats with screening criteria of fold change ≥ 2 and FDR adjusted *p* < 0.05. Volcano plots were generated using the “EnhancedVolcano” package to visualize the DEGs. Heat maps were constructed using

### Protein-protein interaction (PPI) analysis

The online tool STRING^1^ was utilized to investigate the potential PPI network of the DEGs (Szklarczyk et al., 2021). The PPI network was constructed using a confidence score (C) of ≥0.7 as the cut-off criterion. The constructed network was then imported into Cytoscape and further analyzed using the MCC algorithm. Then the Cytoscape’s molecular complex detection (MCODE) plugin was used to screen the PPI network modules with degree cut-off = 2, node score cut-off = 0.2, k-core = 2 and max depth = 100. Further, the Cytohubba plugin was used to explore key genes.

### qRT-PCR

qRT-PCR was performed to detect the expression levels of Ly6g6e in the spinal cord dorsal horn tissue of rats in different groups. Briefly, total RNA was extracted from the tissues using Trizol reagent (15596018, Thermo Fisher, USA). The concentration and purity of the extracted RNA were assessed using a NanoDrop spectrophotometer. Subsequently, cDNA was synthesized using a random primer reverse transcription kit (4374966, Thermo Fisher, USA). The expression levels of the key genes were determined using the SYBR™ Green PCR premix (4309155, Thermo Fisher, USA), following the manufacturer’s instructions. β-actin was used as an internal reference control. The experimental data obtained from quantitative real-time PCR (qRT-PCR) were analyzed using the 2^–ΔΔCt^ method to calculate the relative expression of the target gene. The primer sequences used in the qRT-PCR are provided in Supplementary Table 1.

### Western blot

Total protein of spinal cord dorsal horn tissue of rats was extracted using RIPA lysate (R0010, Solarbio, China). The concentration of the extracted protein was determined using a BCA protein concentration assay kit (enhanced) (P0010S, Beyotime, China). Subsequently, 20 μg of protein was denatured by boiling in 5 × loading buffer. The denatured proteins were separated by SDS-PAGE and transferred to a PVDF membrane. The membrane was then blocked with 5% non-fat dry milk for 2–3 h. Following blocking, the membrane was incubated overnight at 4°C with primary antibodies, including LY-6G/Ly-6E Polyclonal Antibody (PA5-84280, Thermo Fisher, USA), CGRP antibody (PA5-114929, Thermo Fisher, USA), NGF antibody (PA5-29425, Thermo Fisher, USA) and anti-β-actin (ab8226, Abcam, USA). After rinsing the membrane with TBST (Tris-buffered saline with Tween 20) three times, secondary antibodies, such as goat anti-mouse antibody against IgG (ab205719, Abcam, USA) and goat anti-rabbit antibody against IgG (ab6721, Abcam, USA), were added for an additional 1-h incubation at room temperature. After three washes with TBST, the membrane was treated with a chemiluminescence reagent (YA0372, Solarbio, China) and then placed in a gel imaging system for development and image acquisition. The protein bands’ grayscale intensity was analyzed using Image J software, and the relative protein expression was calculated by normalizing it to β-actin, which served as an internal reference.

### Enzyme-linked immunosorbent assay (ELISA)

ELISA detection of IL-1β (YC-30419, Beijing Ilerui Biotechnology Co., Ltd), MCP-1 (ab219045, Abcam, UK), and IL-6 (ab100772, Abcam, UK) levels in the spinal cord dorsal horn tissue of rats from the Sham group, SCN-CCI group, SCN-CCI + siNC group, and SCN-CCI + si-Ly6g6e group was performed. In short, strictly follow the instructions of the reagent kit for the experiment.

### Immunofluorescence staining

This experiment was performed to observe the activation of microglial cells (CD68, ab283654, Abcam, UK) in the spinal cord dorsal horn tissue of rats from the Sham group, SCN-CCI group, SCN-CCI + siNC group, and SCN-CCI + si-Ly6g6e group. In short, tissue samples were fixed with 4% paraformaldehyde. Subsequently, the sample was incubated with 0.3% TritonX-100 containing serum to block the non-specific binding site. Next, CD68-specific primary antibody was added to the sample and incubated at 4°C overnight. The sample was cleaned the next day with PBST to remove unbound primary antibodies. Then, incubate the sample with fluorescently labeled secondary antibodies. The sample was washed again to remove unbound secondary antibodies. Finally, the activation of microglia was observed under fluorescence microscope, and the images were collected and analyzed.

### Statistical analysis

The experiments in this study were all carried out under the condition of blind method. All data were expressed as mean ± standard deviation (SD). Statistical analysis was performed using SPSS 17.0 software. Behavioral assessments of pain, including mechanical allodynia, thermal hyperalgesia, and cold allodynia, were analyzed using repeated measures analysis of variance (ANOVA). Student’s *t*-test was used to analyze the difference between the two groups, and one-way ANOVA or two-way ANOVA was used to compare the difference between multiple groups. Tukey *post hoc* comparison test for multiple comparisons. Differential gene expression analysis was conducted using appropriate statistical methods, such as edgeR, with multiple testing corrections. A significance level of *P* < 0.05 was considered statistically significant.

## Results

### Establishment of SCN-CCI rat model

**FIGURE 1:**
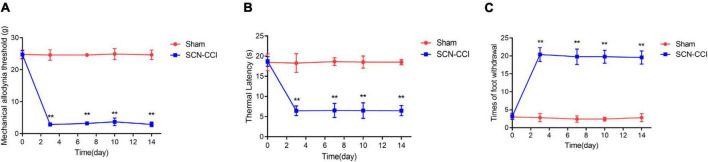
Establishment of sciatic nerve chronic constriction injury (SCN-CCI) rat model (*n* = 6 rats/group). **(A–C)** The mechanical allodynia threshold **(A)**, thermal hyperalgesia latency **(B)** and times of paw withdrawal **(C)** of rats in the Sham and SCN-CCI group measured on days 0, 3, 7, 10, and 14 (*n* = 6 rats), **P < 0.01 *vs*. Sham group. SCN-CCI, chronic constriction injury. Student’s *t*-test is used for data analysis.

This study obtained a rat model of SCN-CCI by ligating the sciatic nerve. Behavioral tests were conducted to assess the effects of SCN-CCI on pain sensitivity. The results revealed significant differences between theSCN-CCI group and the Sham group. Starting from the 3rd day, the SCN-CCI group exhibited lower mechanical allodynia thresholds and thermal latencies than the Sham group, indicating an increased sensitivity to mechanical stimuli and a decreased response time to thermal stimuli in the SCN-CCI group. Moreover, there was a noticeable increase in the number of paw withdrawals in the SCN-CCI group, indicating heightened pain responses. Specifically, the mechanical allodynia threshold remained consistently low at 2–4 g, and the thermal latency remained relatively short at 4–8 s from the 3rd to the 14th day in the SCN-CCI group. Additionally, the number of paw withdrawals remained relatively high throughout this period (Figures 1A–C).

### Screening of DEGs

**FIGURE 2:**
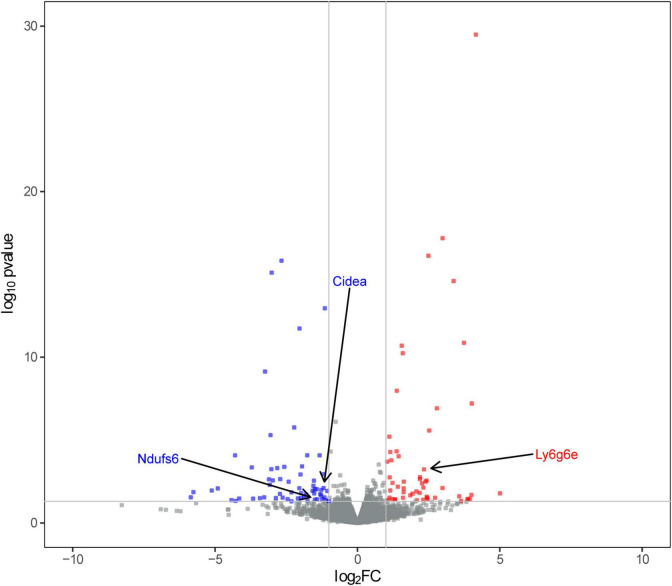
Screening of sciatica-associated differentially expressed genes (DEGs) (*n* = 2 rats/group). Volcano plot of sciatica-associated DEGs; SCN-CCI, sciatic nerve chronic constriction injury.

Subsequently, we obtained RNA-Seq data from left L4-L6 spinal segment tissues. We conducted differential analysis of the sequencing data using the criteria of | log2FC | > 1 and *P* < 0.05. As a result, we identified a total of 117 differentially expressed genes (DEGs), including 55 up-regulated and 62 down-regulated DEGs, in the SCN-CCI rat model (Figure 2). Supplementary Table 2 showed the top 10 up-regulated and down-regulated DEGs.

### Building PPI network modules

**FIGURE 3:**
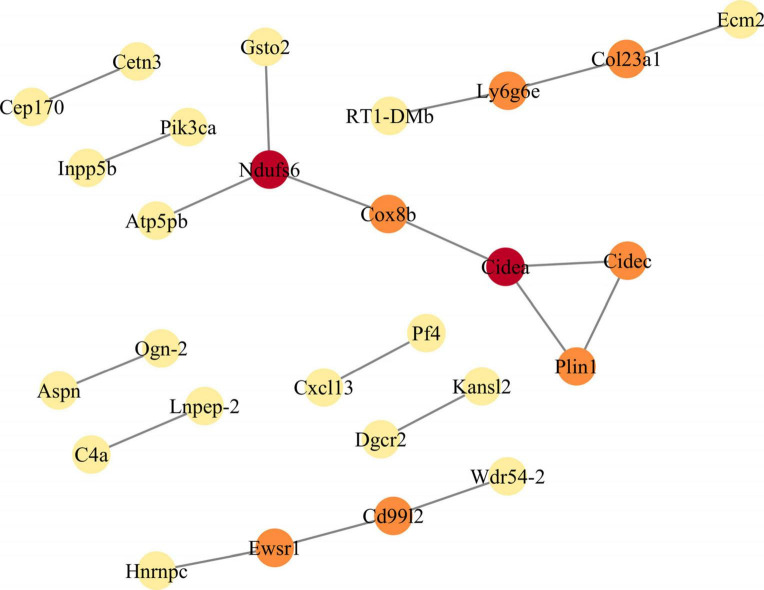
Constructs the protein-protein interaction (PPI) network module of differentially expressed genes (DEGs). The PPI network of DEGs is constructed by STRING database, and the PPI network module is obtained by MCODE plugin in Cytoscape. The shade of color indicates the score size of the node (red > orange > yellow). PPI, protein-protein interaction; DEGs, differentially expressed genes.

To further screen for DEGs, we utilized the STRING database to construct PPI networks, which were subsequently analyzed using Cytoscape to identify hub genes. Based on the screening results, the PPI network modules consisted of 22 protein-protein interaction pairs, comprising 28 nodes and 22 edges. Furthermore, the MCODE plugin ranked the hub genes based on their scores. The highest-scoring DEGs were Ndufs6, Ly6g6e, and Cidea (Figure 3). The expressions of Ndufs6 and Cidea were down-regulated, while the expression of Ly6g6e was up-regulated. Therefore, in the follow-up experiments, we focused on the role of Ly6g6e in SCN-CCI rats.

### Identification of hub genes

**FIGURE 4:**
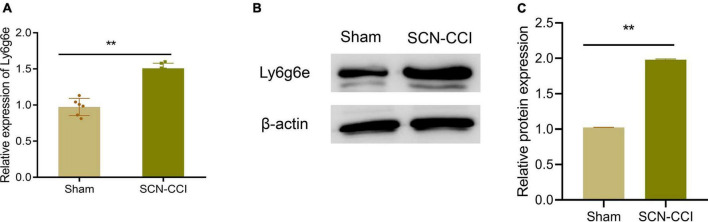
Identification of Hub Gene. **(A)** mRNA levels of Ly6g6e **(A)** in the left L4-L6 spinal cord tissue of the Sham and SCN-CCI groups were detected by qRT-PCR at day 14 (*n* = 6 rats/group); **(B,C)**: protein expression levels of Ly6g6e in the left L4-L6 spinal cord tissue of the Sham and SCN-CCI groups were detected by western blot (*n* = 3 rats/group). **P < 0.01 *vs*. Sham, Sham surgery group; SCN-CCI, chronic constriction injury. Student’s *t*-test is used for data analysis.

Subsequently, qRT-PCR was performed to assess the Ly6g6e transcript levels. The qRT-PCR results demonstrated a significant increase in the mRNA expression of Ly6g6e in rats from the SCN-CCI group compared to the Sham group (Figure 4A). Additionally, Western blot analysis was conducted to examine Ly6g6e protein expression levels. The results showed a substantial increase in the protein expression of Ly6g6e in the SCN-CCI group rats compared to the Sham group rats (Figures 4B, C). These experimental findings were consistent with the results obtained from the RNA-Seq sequencing, confirming the differential expression patterns of Ly6g6e in the SCN-CCI rat model.

### Changes Ly6g6e expression levels in the spinal dorsal horn tissues in NP rat models

**FIGURE 5:**
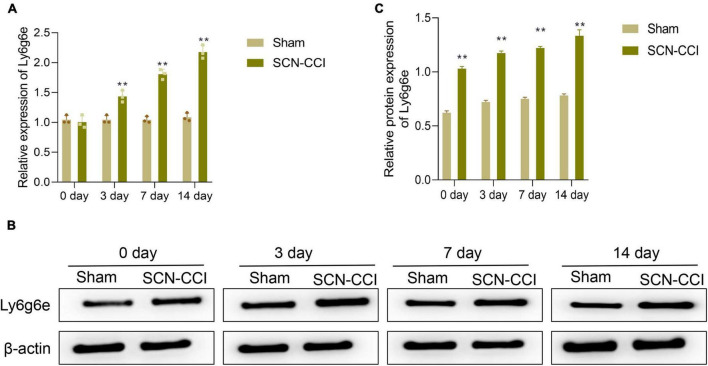
After modeling, the expression level of Ly6g6e in the spinal dorsal horn tissue of rats was observed. **(A)** The mRNA expression level of Ly6g6e in spinal dorsal horn tissues of rats in different groups was detected by qRT-PCR at day 0, 3, 7 and 14 (*n* = 3 rats/group). **(B,C)** Western blot was used to detect Ly6g6e protein expression levels at day 0, 3, 7 and 14 in spinal dorsal horn tissues of rats in different groups (*n* = 3 rats/group). **P < 0.01 *vs*. Sham group. Sham, Sham surgery group; SCN-CCI, chronic constriction injury. Student’s *t*-test is used for data analysis.

At 0 days, we found no significant difference in the expression levels of Ly6g6e in the spinal dorsal horn tissue between the Sham and SCN-CCI groups of rats. However, at 3, 7, and 14 days, compared to the Sham group, the SCN-CCI group exhibited a significant increase in the expression levels of Ly6g6e in the spinal dorsal horn tissue, which further increased with time (Figures 5A–C).

**FIGURE 6:**
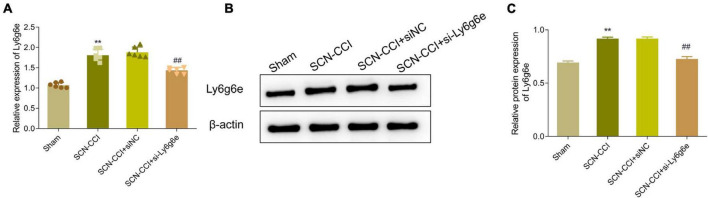
Expression level of Ly6g6e siRNA in SCN-CCI rats. **(A)** The mRNA expression level of Ly6g6e in spinal dorsal horn tissues of rats in each group was detected by qRT-PCR at day 14 (*n* = 6 rats/group). **(B,C)** Western blot analysis of Ly6g6e protein expression in spinal dorsal horn tissues of rats in different groups at day 14 (*n* = 3 rats/group), **P < 0.01 *vs*. Sham group. ##*P* < 0.01 *vs*. SCN-CCI + siNC group. ANOVA followed Tukey *post hoc* test is used for data analysis.

Additionally, compared to the SCN-CCI + siNC group, the SCN-CCI + si-Ly6g6e group rats demonstrated a significant downregulation in the expression levels of Ly6g6e in the spinal dorsal horn tissue. However, there were no statistically significant differences observed between the SCN-CCI group and the SCN-CCI + siNC group (Figures 6A–C).

### Pain behavior assessment following Ly6g6e siRNA treatment in the SCN-CCI rat models

*(1) PWMT analysis and effects of Ly6g6e siRNA treatment in the SCN-CCI rat models*

**FIGURE 7:**
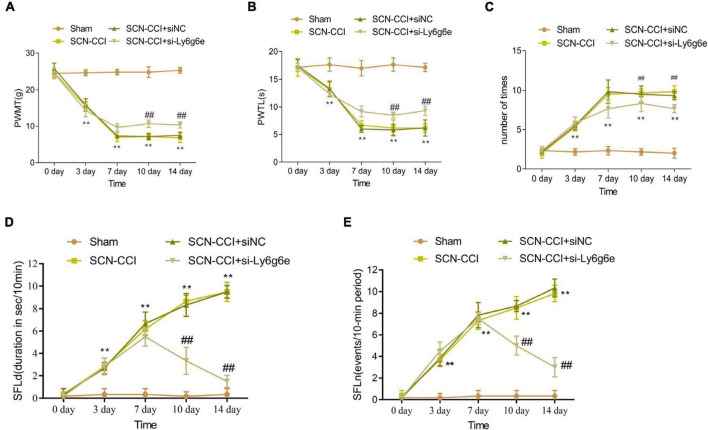
The effect of Ly6g6e siRNA on pain behavior in SCN-CCI rats. **(A–C)** The induced pain behavior of rats in the sham group, SCN-CCI group, SCN-CCI + siNC group, and SCN-CCI + si-Ly6g6e group was evaluated at day 0, 3, 7, 10, and 14 (*n* = 6 rats/group), including: **(A)** mechanical dysodynia (mechanical threshold for claw withdrawal, PWMT), **(B)** thermal hypersensitivity (latent period of claw deheating, PWTL), **(C)** cold dysodynia. **(D,E)** The spontaneous pain behaviors of rats in the sham group, SCN-CCI group, SCN-CCI + siNC group, and SCN-CCI + si-Ly6g6e group was evaluated at day 0, 3, 7, 10, and 14 (*n* = 6 rats/group), including: **(D)** spontaneous foot-lifting (SFL) duration (SFLd), **(E)** spontaneous foot-lifting (SFL) number (SFLn),**P < 0.01 *vs*. Sham group. ##*P* < 0.01 *vs*. SCN-CCI + siNC group. Two-way ANOVA followed Tukey *post hoc* test is used for data analysis.

Over the course of 0, 3, and 7 days, the PWMT showed a decreasing trend in the SCN-CCI group, SCN-CCI + siNC group, and SCN-CCI + si-Ly6g6e group. However, from 10 to 14 days, the PWMT in these groups fluctuated within a certain range. At 7, 10, and 14 days, compared to the Sham group, the PWMT of the SCN-CCI group was significantly decreased. Furthermore, compared to the SCN-CCI + siNC group, the PWMT of the SCN-CCI + si-Ly6g6e group was significantly increased. In addition, there were no statistically significant differences between the SCN-CCI group and the SCN-CCI + siNC group. The PWMT of the Sham group remained within the normal range of 23–26 g throughout 0, 3, 7, 10, and 14 days (Figure 7A).

*(2) PWTL analysis and effects of Ly6g6e siRNA treatment in the SCN-CCI rat models*

Over the course of 0, 3, and 7 days, the PWTL showed a decreasing trend in the SCN-CCI group, SCN-CCI + siNC group, and SCN-CCI + si-Ly6g6e group. However, from 10 to 14 days, the PWTL in these groups fluctuated within a certain range. At 7, 10, and 14 days, compared to the Sham group, the PWTL of the SCN-CCI group was significantly decreased. Additionally, compared to the SCN-CCI + siNC group, the PWTL of the SCN-CCI + si-Ly6g6e group was significantly increased. Of note, there were no statistically significant differences between the SCN-CCI group and the SCN-CCI + siNC group. The PWTL of the Sham group remained within the normal range of 16–19 s throughout 0, 3, 7, 10, and 14 days (Figure 7B).

*(3) Cold Stimulation-Evoked Paw Flinching evaluation and effects of Ly6g6e siRNA treatment in the SCN-CCI rat models*

Comparatively, the frequency of paw flinches in the SCN-CCI group, SCN-CCI + siNC group, and SCN-CCI + si-Ly6g6e group showed an increasing trend from 0 to 3 and 7 days and then fluctuated within a certain range from 10 to 14 days. At 7, 10, and 14 days, compared to the Sham group, the SCN-CCI group showed a significant increase in the number of paw flinches. Additionally, compared to the SCN-CCI + siNC group, the SCN-CCI + si-Ly6g6e group showed a significant decrease in the number of paw flinches. However, there were no statistically significant differences between the SCN-CCI group and the SCN-CCI + siNC group. The number of paw flinches in the Sham group remained within the normal range throughout 0, 3, 7, 10, and 14 days (Figure 7C).

*(4) Effect of Ly6g6e siRNA on spontaneous pain behavior in SCN-CCI rat models.*

The results showed that at day 0, there were no significant differences in SFLn and SFLd among the Sham group, SCN-CCI group, SCN-CCI + siNC group, and SCN-CCI + si-Ly6g6e group rats. The SFLn and SFLd of Sham group rats remained at relatively low levels at 0, 3, 7, 10, and 14 days. The SFLn and SFLd of SCN-CCI group and SCN-CCI + siNC group rats showed an increasing trend at 0, 3, 7, 10, and 14 days. The SFLn and SFLd of the SCN-CCI + si Ly6g6e group showed an upward trend at 0, 3, and 7 days, reaching their peak at 7 days, and then showing a slow downward trend. Compared with the SCN-CCI + siNC group, the SFLn and SFLd of the SCN-CCI + si-Ly6g6e group rats were significantly reduced at 7, 10, and 14 days, and slightly decreased at 3 days, but the difference was not statistically significant. (Figures 7D, E).

### Alterations in CGRP and NGF protein expression levels in the spinal dorsal horn tissue during NP development

**FIGURE 8:**
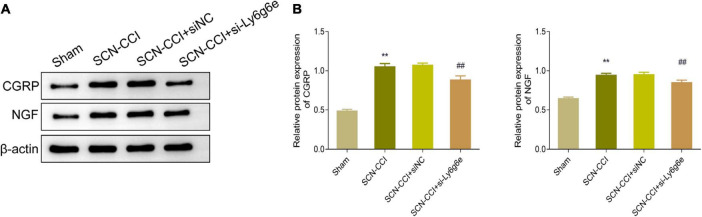
Knocking down Ly6g6e inhibits the protein expression levels of CRPG and NGF in the spinal dorsal horn tissue of SCN-CCI rats. **(A,B)** Western blot was used to detect the protein expression levels of CRPG and NGF in spinal dorsal horn tissues of rats in Sham group, SCN-CCI group, SCN-CCI + siNC group and SCN-CCI + si-Ly6g6e group at day 14 (*n* = 3 rats/group). **P < 0.01 *vs*. Sham group. ##*P* < 0.01 *vs*. SCN-CCI + siNC group. ANOVA followed Tukey *post hoc* test is used for data analysis.

In regard to the protein levels of CGRP and NGF in the spinal dorsal horn tissues, compared to the Sham group, the SCN-CCI group rats exhibited a significant increase in these proteins. Conversely, compared to the SCN-CCI + siNC group and the SCN-CCI + si-Ly6g6e group, we observed a significant decrease in CGRP and NGF protein levels in the spinal dorsal horn tissue. However, we observed no statistically significant differences between the SCN-CCI group and the SCN-CCI + siNC group (Figures 8A, B).

### Activation of microglial cells in the spinal dorsal horn

**FIGURE 9:**
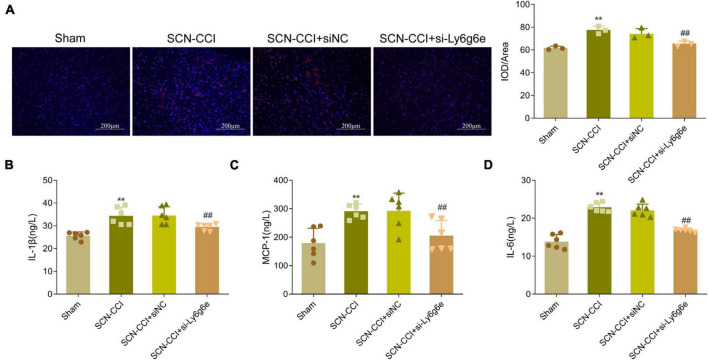
Knockdown of Ly6g6e inhibits the secretion of inflammatory factors and the activation of Microglia in SCN-CCI rats. **(A)** The activation of microglial cells (CD68) in spinal dorsal horn tissues of rats in Sham group, SCN-CCI group, SCN-CCI + siNC group and SCN-CCI + si-Ly6g6e group was observed by immunofluorescence staining at day 14. **(B–D)** ELISA detection of IL-1β, MCP-1, and IL-6 levels in the spinal cord dorsal horn tissue of rats from the Sham group, SCN-CCI group, SCN-CCI + siNC group, and SCN-CCI + si-Ly6g6e group at day 14 (*n* = 6 rats/group). **(B–D)** Bar graphs showing the comparison of panel **(B)** IL-1β, **(C)** MCP-1 and **(D)** IL-6 levels in the spinal dorsal horn tissue between different experimental groups. Data are presented as mean ± standard deviation. **P < 0.01 *vs*. Sham group. ##*P* < 0.01 *vs*. SCN-CCI + siNC group. ANOVA followed Tukey *post hoc* test is used for data analysis.

Compared to the Sham group, the spinal dorsal horn of the SCN-CCI group rats exhibited a significant activation of microglial cells, accompanied by a substantial increase in microglial cells. Conversely, compared to the SCN-CCI + siNC group, the SCN-CCI + si-Ly6g6e group rats demonstrated a significant reduction in the activation of microglial cells, leading to a significant decrease in the number of microglial cells. However, we observed no statistically significant differences between the SCN-CCI group and the SCN-CCI + siNC group (Figure 9A).

### IL-1β, MCP-1, and IL-6 levels in the spinal dorsal horn

Compared to the Sham group, the levels of IL-1β, MCP-1, and IL-6 in the spinal dorsal horn of the SCN-CCI group rats were significantly increased. In contrast, compared to the SCN-CCI + siNC group, the SCN-CCI + si-Ly6g6e group rats exhibited a significant decrease in IL-1β, MCP-1, and IL-6 levels. However, no statistically significant differences were observed between the SCN-CCI group and the SCN-CCI + siNC group (Figures 9B–D).

## Discussion

In this study, we successfully established a rat model of SCN-CCI by ligating the sciatic nerve to induce chronic constriction injury mimicking the pathological changes observed in human sciatica and NP conditions. The observed behavioral changes in the SCN-CCI group, including lower mechanical allodynia thresholds, decreased thermal latencies, and increased paw withdrawals and spontaneous pain behaviors in response to cold pain sensation, are indicative of heightened pain sensitivity and reflect the characteristic symptoms of NP and also align with previous studies conducted by Moini-Zanjani et al. (2016), Sun et al. (2020), Banik et al. (2023), who demonstrated that SCN-CCI surgery led to a significant decrease in the mechanical withdrawal threshold and paw thermal withdrawal latency, along with an increased response rate to cold acetone. Therefore, the successful establishment of the SCN-CCI rat model in this study provides a relevant and reliable model for investigating genes associated with NP, related underlying mechanisms and potential therapeutic interventions.

The elevated levels of IL-1β, MCP-1, and IL-6 in the spinal dorsal horn of the SCN-CCI group are consistent with the involvement of neuroinflammation in NP. These cytokines have been implicated in mediating glial activation, neuroinflammatory responses, and the sensitization of pain pathways (Hester et al., 2022; Damiati et al., 2023; Paranga et al., 2023), and the increased activation of microglial cells observed in the SCN-CCI group further supports the role of neuroinflammation in NP pathophysiology. Microglial activation has been associated with releasing pro-inflammatory mediators and amplifying pain signaling (Dheen et al., 2007; Smith et al., 2012). The findings from this study align with previous research highlighting the contribution of neuroinflammatory processes in NP models (Cedeno et al., 2021; Dalenogare et al., 2023). Therefore, to a certain extent, our findings suggest that targeting neuroinflammation and its associated cytokines, as well as modulating microglial activation, could be a promising approach for alleviating NP.

The field of biotechnology, encompassing molecular biology, high-throughput sequencing, and bioinformatics, has received increasing attention for its potential to identify new therapeutic targets for various diseases. Chen et al. (2023) sequenced single cell RNA of rat dorsal root ganglion neurons and found that adcyap1 can act as a protective factor connecting pain and nerve regeneration. At the same time, Du et al. (2023) used single-cell RNA sequencing (scRNA-seq) to identify age-related molecular responses in spinal astrocytes under neuropathic pain and identified four potential age-related genes in the spinal astrocyte population. It can be seen that RNA sequencing is of great significance in studying the mechanism of disease. In this study, we utilized RNA-Seq to obtain expression profiles in the left L4-L6 spinal segments of rats in the SCN-CCI group and rats in the Sham group. The identification of 117 DEGs in the SCN-CCI rat model provides valuable insights into the molecular alterations underlying NP. The identified DEGs were found to exhibit molecular functions such as CXCR chemokine receptor binding, receptor regulator activity during signal transmission, and transmembrane transporter activities. They were also localized in cellular components such as methylosomes, MHC class II protein complexes, and lipid droplets. The enrichment analysis revealed the involvement of these DEGs in various biological processes and pathways related to lipid catabolism, IMP metabolism, histone phosphorylation, and negative regulation of tumor necrosis factor. Additionally, the pathways associated with staphylococcus aureus infection, primary bile acid biosynthesis, and β-alanine metabolism were significantly enriched. Previous studies have confirmed that β-alanine metabolism can increase calcium levels in neurons of the dorsal root ganglion, leading to pain behaviors in the SCN-CCI group (Xiao X. et al., 2019), indicating the potentially important role of β-alanine metabolism in the development of NP. In addition, although lipid catabolism has been implicated as a relevant factor in neuronal death after traumatic brain injury (Xiao X. et al., 2019), its specific impact on NP has not been thoroughly investigated or confirmed. Further, while some of these pathways have been previously implicated in NP (Iqbal et al., 2021; Navia-Pelaez et al., 2021; Xu et al., 2022), the specific roles of the identified DEGs in these pathways remain to be elucidated.

Through differential analysis and protein-protein interaction (PPI) network analysis, we identified Ly6g6e as key differentially expressed genes (DEGs) in the SCN-CCI group. The upregulation of Ly6g6e expression in the spinal dorsal horn of the SCN-CCI group is a novel finding in the context of NP. While the specific role of Ly6g6e in NP has not been extensively studied, its upregulation suggests its potential involvement in the pathogenesis of NP. Consistent with the RNA-Seq findings, our subsequent experiments using RT-PCR and Western blot techniques confirmed that Ly6g6e was significantly up-regulated in the spinal tissues of SCN-CCI rats. Previous literature indicated that Ly6g6e, a member of the Ly-6 superfamily, is a cell surface protein involved in cell adhesion and migration, with high expression at the leading edge of cells (Mallya et al., 2006). Lymphocytes have been closely associated with NP development, as they accumulate in injured peripheral nerves and contribute to acute inflammatory responses and the release of neurotrophic factors, leading to acute pain responses (Galvin and C, 2021). Especially in intractable neuropathic pain, when peripheral nerves are damaged, the damaged axons develop Waller’s degeneration. Schwann cells, mast cells and epithelial cells are activated to release cytokines, chemokines and growth factors, forming an inflammatory cascade. These primary mediators sensitize sensory nerve endings, and then recruit macrophages, neutrophils and lymphocytes to the site of inflammation, causing changes in gene expression and promoting post-translation modification of proteins. And change the ion channel function of primary afferent neurons. This results in increased excitability and spontaneous activity, and the production of secondary mediators, including colony-stimulating factor 1 (CSF-1), chemokine C-C motile ligand 21 (CCL-21), Wnt3a, and Wnt5a. The release of these mediators from primary afferent neurons alters the properties of spinal microglia, causing them to release tertiary mediators such as BDNF, tumor necrosis factor α (TNF-α), interleukin-1β (IL-1β) and other Wnt ligands by increasing excitatory glutamatergic transmission in the dorsal horn of the spinal cord and decreasing inhibitory GABA and glycinergic transmission. Promote the generation and transmission of injurious information, which leads to the aggravation of neuropathic pain (Boakye et al., 2021). Hence, the elevated expression of Ly6g6e in SCN-CCI rats may be related to lymphocyte migration, and considering the observed activation of microglial cells in the SCN-CCI group, it is plausible that Ly6g6e may play a role in neuroinflammatory processes associated with NP. This well explains our results. In this study, we found that siRNA Ly6g6e can significantly inhibit neuroinflammation and microglia activation in SCN-CCI rats, and at the same time, reduce neuropathic pain.

## Conclusion

In conclusion, the findings from this study contribute to our understanding of the molecular mechanisms underlying NP. Establishing the SCN-CCI rat model and characterizing pain-related behaviors confirm its suitability for studying NP processes. The differential expression of Ly6g6e, along with the activation of microglial cells and elevated cytokine levels, suggests its potential role in neuroinflammation and pain sensitization. The identification of DEGs and their enrichment in relevant biological processes and pathways provide new avenues for exploring NP mechanisms. Furthermore, the hub genes Ly6g6e offer potential targets for further investigation and the development of novel therapeutic strategies. Overall, these findings contribute to the advancement of knowledge in NP research and could be used as a referential basis for developing more effective treatments for individuals suffering from this condition.

## Data availability statement

The datasets generated and/or analysed during the current study are available in BioProject: PRJNA1071321 (https://www.ncbi.nlm.nih.gov/bioproject/?term=PRJNA1071321).

## Ethics statement

The animal studies were approved by the First Affiliated Hospital of Nanchang University (CDYFY-IACUC-202305QR014). The studies were conducted in accordance with the local legislation and institutional requirements. Written informed consent was obtained from the owners for the participation of their animals in this study.

## Author contributions

MX: Conceptualization, Investigation, Resources, Writing – review and editing. ZW: Methodology, Writing – original draft. GX: Project administration, Writing – original draft. MZ: Conceptualization, Writing – review and editing. DZ: Data curation, Formal analysis, Writing – review and editing. YY: Data curation, Formal analysis, Investigation, Writing – review and editing.

## Footnotes

1
https://string-db.org/
