# Supplementary material for: Exploring gene signatures and regulatory networks in a rat model of sciatica: implications and validation in neuropathic pain
Source: Front Mol Neurosci. 2024 Feb 6;16:1261217. doi: 10.3389/fnmol.2023.1261217 (PMC10877633; doi:10.3389/fnmol.2023.1261217)
Supplement: Supplementary file 1 [file Table_1.DOCX]

Supplementary Table 1 Primers for qRT-PCR

| Genes | Sequences (5' to 3') |
| --- | --- |
| Ly6g6e | F:CAGCAACTCCTGCAAGCAGACA |
|  | R:CTGGGTAGTGATGGCTCAAGGT |
| Aurkb | F:CTTCTACGACCAGCAGAGGATC |
|  | R:GGCATCTGACAGTTCCTCCATG |
|  | R:TGATGATGCTGTTTCCTCGGATG |
| Cidea | F:GGTGGACACAGAGGAGTTCTTTC |
|  | R:CGAAGGTGACTCTGGCTATTCC |
| β-actin | F: TCAAGATCATTGCTCCTCCTGAG |
|  | R: ACATCTGCTGGAAGGTGGACA |

Supplementary Table 2 The first 10 up-regulated and down-regulated genes

| Genes | Style |
| --- | --- |
| AABR07001512.1 | up |
| Aurkb | up |
| Ly6g6e | up |
| Cidea | down |
| LOC108348139 | up |
| Nhp2 | down |
| Snrpg | up |
| Ibsp | up |
| RT1-DMb2 | down |
| Cidec | down |
| Plin1 | down |
| NEWGENE_1308171 | down |
| Cxcl13 | up |
| Ly6g6d | up |
| Pf4 | up |
| LOC100910978 | down |
| LOC100911576 | up |
| LOC100912481 | down |
| Enpp6 | up |
| Echs1 | down |
